# Vitamin D levels and oxidative stress markers in patients hospitalized with COVID-19

**DOI:** 10.1080/13510002.2021.1999126

**Published:** 2021-11-02

**Authors:** Emilija Atanasovska, Marija Petrusevska, Dragica Zendelovska, Katerina Spasovska, Milena Stevanovikj, Katerina Kasapinova, Kalina Gjorgjievska, Nikola Labachevski

**Affiliations:** a Faculty of Medicine, University of Ss Cyril and Methodius, Institute of Preclinical and Clinical Pharmacology and Toxicology, Skopje, Republic of North Macedonia; b Intensive Care Unit, University Clinic for Infectious Diseases and Febrile Conditions, Skopje, Republic of North Macedonia; c Intensive Care Unit, University Surgery Clinic ‘St.Naum Ohridski', Skopje, Republic of North Macedonia

**Keywords:** COVID-19, vitamin D, oxidative stress, plasma peroxides, disease severity, antioxidants, inflammation

## Abstract

**Background:**

COVID-19 is characterized by the presence of oxidative stress. Vitamin D status has been reviewed as one of the factors that may affect disease severity. The aim of this study was to assess the relationship between serum vitamin D levels, oxidative stress markers and disease severity in hospitalized COVID-19 patients.

**Methods:**

Vitamin D levels were measured in 33 patients with COVID-19. The total antioxidant power and plasma peroxides were determined in serum.

**Results:**

Severe COVID-19 patients have lower vitamin D levels (18.39 ± 2.29 ng/mL vs. 28.47 ± 3.05 ng/mL, *p* < .05) and higher oxidative stress compared to the moderate group. When divided according to serum vitamin D levels, significantly higher values of LDH (604.8 ± 76.98 IU/mL vs. 261.57 ± 47.33 IU/mL) and D-dimer (5978 ± 2028ng/mL vs. 977.7 ± 172 ng/mL) were obtained in the group with vitamin D below 30 ng/mL, followed with significantly higher levels of plasma peroxides (d-ROMs: 414.9 ± 15.82 U.Carr vs. 352.4 ± 18.77 U.Carr; *p* < .05) and oxidative stress index (OSI: 92.25 ± 6.60 vs. 51.89 ± 6.45; *p* < .001).

**Conclusion:**

The presented data provide a justification to consider vitamin D as an important factor that could ameliorate disease severity through its anti-inflammatory and antioxidant effects.

## 1. Introduction

COVID-19 is an acute respiratory infection caused by the novel SARS-CoV- 2 virus and so far has caused more than 241.5 million confirmed cases and more than 4.9 million deaths worldwide [1,2]. It is well established that most of the patients are asymptomatic or manifest a mild form of the disease, but however, 15% require hospitalization and pose a major burden to the healthcare systems. Although the number of COVID-19 related scientific papers is substantial, the pathophysiologic mechanisms underlying the disease are not completely elucidated and the clinicians still need additional, easily accessible and affordable prognostic markers for differentiation of patients that might develop a more serious form of the disease and worse outcome.

Vitamin D is a fat-soluble vitamin that is responsible for the regulation of calcium and phosphate metabolism and maintaining a healthy mineralized skeleton, but it is also known as an immunomodulatory hormone [3–6]. Vitamin D deficiency has been associated with increased morbidity and mortality from respiratory tract infections, as well as a higher incidence of developing acute respiratory distress syndrome [7–9].

D’Avolio et al. [10] reported that significantly lower 25(OH)D levels were measured in PCR-positive for SARS-CoV-2 patients compared with negative patients and proposed that vitamin D supplementation might be a useful measure in the prevention of this infection. Association between low serum vitamin D levels and increased risk of developing severe COVID-19 has been noted [11, 12], but it is still not clear if there are any other effects of vitamin D that might influence the host response to the SARS-CoV-2 infection in addition to its immunomodulatory action. De las Heras et al. [13] suggest that vitamin D could also be involved in the regulation of oxidative stress in COVID-19. Several review articles point to the oxidative stress as a potential key player in SARS-CoV-2 infection [14–17]. Cecchini proposes a hypothesis that oxidative stress is associated with the changes found in COVID-19 patients, such as cell hypoxia, coagulopathy and the cytokine storm [18]. Therefore, the measurement of oxidative stress markers in a clinical setting could be a valuable tool to determine the progress of COVID-19 [19].

Clinical data on vitamin D status in conjunction with oxidative stress parameters in patients infected with SARS-Cov-2 are limited. Our study intends to deepen the knowledge about vitamin D status in relation to oxidative stress parameters measured by fast and inexpensive analytical photometric method in patients infected with SARS-CoV-2.

The aim of this study was to assess the potential relationship between serum vitamin D levels, oxidative stress parameters and disease severity in hospitalized COVID-19 patients.

## 2. Material and methods

### 2.1. Study population

Thirty-three COVID-19 patients hospitalized at the University Clinic for Infectious diseases and Febrile Conditions, Skopje, Republic of North Macedonia within a period of one week during the winter season and active lockdown within the last six months were included. All patients in the study group were hospitalized with reduced sun exposure. All patients were confirmed to have SARS-CoV-2 infection by real-time reverse transcriptase-polymerase chain reaction assay (RT–PCR) from nasopharyngeal swab specimen. The study was approved by the Ethics Committee of the Faculty of Medicine, University of Ss Cyril and Methodius, Skopje, Republic of North Macedonia, No #03-366/7.

The diagnosis and classification of COVID-19 were based on the Interim Guidance for Clinical Management of COVID-19 issued by the WHO (2). Patients with moderate disease were adults with clinical signs of pneumonia, but no signs of severe pneumonia, including SpO_2_ ≥ 90% on room air. Severe cases additionally met at least one of the following conditions: SpO_2_ < 90% on room air, respiratory rate >30 breaths/minute or presence of severe respiratory distress. COVID-19 patients were also divided according to the serum vitamin D levels into two groups: vitamin D insufficient (<30 ng/mL) and sufficient (>30 ng/mL) as per international recommendations [20, 21].

### 2.2. Clinical characteristics and laboratory data

Demographic characteristics, medical history, clinical symptoms and signs, concomitant medication, outcome data, as well as laboratory analyzes were obtained from the patients’ medical records. The selected laboratory parameters that were analysed for the purpose of this study were: blood cell count, neutrophil to lymphocyte ratio (NLR), C reactive protein (CRP), creatine kinase (CK), lactate dehydrogenase (LDH) and D-dimer. Serum vitamin D levels were measured as 25(OH)D at hospital admission.

### 2.3. Method for determination of d-ROMs, PAT and oxidative stress index

PAT (total antioxidant power, iron reducing) and d-ROMs (plasma peroxides) were measured on FRAS5 analytical photometric system (H&D, Parma, Italy). The instructions of the manufacturer were followed for the both tests. Serum samples were taken on admission at the clinic. The d-ROM and PAT are reported in equivalents of H_2_O_2_ and ascorbic acid, respectively. The d-ROM and PAT reference normal values are 250–300 U. Carr (1 U. Carr = 0.08 mg H_2_0_2_/dL) and 2200–2800 U. Carr, respectively. OSI presents information obtained from d-ROMs Fast test and the PAT test that is automatically calculated by manufacturer’s software with normal reference values less than 40.

### 2.5. Statistical analysis

Data were described as number and/or percentage, range or mean or standard error of mean (SEM), where appropriate. Differences between groups were explored using the *t*-test and Mann–Whitney where appropriate. Linear regression was performed to calculate the correlation between the concentration of Vitamin D and d-ROM, OSI, LDH and D-dimers. A *p*-value <.05 was considered significant. For the purpose of comparison obtained results for both groups, we have used normal oxidative stress values of healthy individuals from our previous measurements. All analyses were made using the statistical program GraphPad 9 (USA).

## 3. Results

### 3.1. Demographics and clinical characteristics of patients

The baseline characteristics among the 33 patients are presented in Table 1. The mean age of the patients was 56.36 ± 12.29 years. The Body Mass Index (BMI) was within normal range (18.5–25 kg/m^2^) in most of the patients (*n* = 20), 6 patients were overweight (range 25–30 kg/m^2^) and 7 were obese (BMI > 30 kg/m^2^). The average time from onset of symptoms to hospital admission was 10.45 ± 2.21 days. Regarding disease severity, 18 patients were classified as moderate and 15 as severe cases.

**Table 1.** Baseline characteristics of the COVID-19 patients**.**

	Study population (*n* = 33)
Gender (Male/ Female)	17/16
Age (year) (mean ± SD)	56.36 ± 12.29
**COVID-19 severity (%)**	
Moderate	18 (54.5%)
Severe	15 (45.5%)
Patients with medical conditions (%)	21 (63.6%)
**Co-existing medical conditions (%)**	
Hypertension	11 (33.3%)
Diabetes	8 (24.2%)
Coronary artery disease	4 (12.1%)
Haematological disease	2 (6.1%)
Benign prostatic hyperplasia	3 (9.1%)
Thyroid disease	2 (6.1%)
Chronic heart failure	1 (3%)
Arrythmia	1 (3%)
Chronic gastrointestinal disease	2 (4%)
**Signs and symptoms at admission (%)**	
High body temperature	25 (75.8%)
Malaise (fatigue)	22 (66.7%)
Tachycardia	20 (60.6%)
Cough	17 (51.5%)
Dyspnoea	15 (45.5%)
Loss of appetite	5 (15.2%)
Nausea	4 (12.1%)
Myalgia	3 (9.1%)
Headache	2 (6.1%)
Diarrhoea	2 (6.1%)
Rhinorrhea	1 (3.0%)

Most of the patients (63.6%) presented at least one underlying medical condition at admission, and the average number of comorbidities was 1.71 ± 0.56 (range 1–3).

### 3.2. Study parameters in COVID-19 patients divided by disease severity

The mean values of the laboratory parameters upon admission are presented in Table 2. The inflammatory markers (CRP, D-dimer, and NLR) were higher and the changes in the blood cell count were more pronounced among the severe COVID- patients compared to the moderate group.

**Table 2.** Laboratory parameters in the moderate and severe group of COVID-19 patients.

	Moderate group (n=18) Mean ± SEM	Severe group (n=15) Mean ± SEM	*p* (Mann–Whitney, *t*-test)
CRP (mg/L)	41.61 ± 9.45	113.9 ± 30.58	.0159
LDH (IU/mL)	266.4 ± 24.10	805.0 ± 88.70	<.0001
CK (U/L)	89.89 ± 17.78	250.3 ± 75.44	.0671
D-dimer (ng/mL)	860.4 ± 83.49	8838 ± 3032	<.0001
RBC (×10^3^µL)	4517 ± 89.47	4772 ± 121.8	.0252
Hemoglobin (g/L)	128.8 ± 2.46	140.8 ± 3.36	.0053
Hematocrit	0.38 ± 0.01	0.41 ± 0.01	.0103
WBC (×10^3^µL)	7.17 ± 0.89	13.74 ± 1.52	.0002
PLT (×10^3^µL)	328.7 ± 25.96	287.3 ± 20.82	.2327
NLR	5.33 ± 1.53	17.84 ± 3.16	.0001
Neutrophils (%)	0.65 ± 0.03	0.89 ± 0.01	.0001
Lymphocytes (%)	0.24 ± 0.03	0.063 ± 0.01	.0001
d-ROM (U.Carr)	351.8 ± 14.76	453.1 ± 13.51	<.0001
PAT (U.Carr)	2754 ± 181	2679 ± 95.37	.8795
OSI	61.33 ± 4.84	105.1 ± 8.41	<.0001
Vitamin D (ng/mL) measured as 25(OH)D.	28.47 ± 3.05	18.39 ± 2.29	.0118

Serum vitamin D levels were significantly higher in the moderate group compared with the values in the severe group of COVID-19 patients (28.47 ± 3.05 ng/mL vs. 18.39 ± 2.29 ng/mL, *p* ≤ .05). Considering the disease severity, more than half (*n* = 11, 61.1%) of the moderate COVID-19 patients and the majority of the severe patients (*n* = 13, 86.7%) were vitamin D insufficient. Significant increase between the moderate and severe group of patients was observed for d-ROM and OSI (*t*-test, *p* < .0001). Additionally, when these parameters were compared with normal values, which are in the range of the normal reference values by the manufacturer (d-ROM = 271 ± 5.590 U.Carr, OSI = 21 ± 2.527, PAT = 2406 ± 71.55 U.Carr), both moderate and severe patients had increased level of d-ROM and OSI (*t*-test, *p* < .0001).

The analysis of the hospital discharge records has shown that all patients in the moderate group have recovered from the disease, whereas in the severe group, 4 patients recovered, and the remaining 11 died.

### 3.3. Study parameters in COVID-19 patients divided by serum vitamin D levels

Vitamin D levels below 30 ng/mL were measured in 24 patients (73%), and the remaining 9 (27%) had vitamin D levels greater than 30 ng/mL. Among the patients with vitamin D levels below 30 ng/mL, 10 of them had insufficiency of vitamin D in the range of 20–30 ng/mL (24.34 ± 1.043 ng/mL), and 14 had deficiency of vitamin D below 20 ng/mL (13.56 ± 0.8825 ng/mL). Due to the small number of patients for further analysis, insufficiency and deficiency group, were joined together. In the comparison of the patients with vitamin D below 30 ng/mL to those with vitamin D greater than 30 ng/mL, the values of LDH (604.8 ± 76.98 IU/mL vs. 261.57 ± 47.33 IU/mL, *t*-test, *p* < .05) and the D-dimer (5978 ± 2028ng/mL vs. 977.7 ± 172 ng/mL, *t*-test, *p* < .05) were significantly higher. Additionally, the patients with vitamin D below 30 ng/mL tended to have higher CRP levels at admission, lower platelet count and higher neutrophil to lymphocyte ratio (NLR) compared to the patients with vitamin D sufficient levels, but these differences did not reach statistical significance (*p* > .05 for all three parameters) (Table 3).

**Table 3.** Laboratory parameters in COVID-19 patients divided by serum Vitamin D measured as 25(OH)D.

	Vitamin D < 30 ng/mL (n=24) Mean ± SEM	Vitamin D >30 ng/mL (*n* = 9) Mean ± SEM	*p* (Mann–Whitney, *t*-test)
Vitamin D (ng/mL)	18.05 ± 1.289	39.46 ± 3.472	**.0001**
CRP (mg/L)	83.29 ± 20.02	51 ± 18.91	.1957
LDH (IU/mL)	604.8 ± 76.98	261.57 ± 47.33	.**0138**
CK (U/L)	150.4 ± 35.46	194.7 ± 105.3	.5750
D-dimer (ng/mL)	5978 ± 2028	977.7 ± 172	.**0429**
RBC (×10^3^µL)	4640 ± 98.93	4614 ± 98.67	.5573
Hemoglobin (g/L)	133.5 ± 2.81	136.0 ± 3.86	.6984
Hematocrit	0.3917 ± 0.008	0.4178 ± 0.019	.3722
WBC (×10^3^µL)	10.67 ± 1.272	9.033 ± 1.501	.6692
PLT (×10^3^µL)	285.2 ± 90	365.8 ± 32.49	.0839
NLR	12.08 ± 2.509	8.178 ± 2.714	.4799
Neutrophils (%)	0.788 ± 0.032	0.72 ± 0.0585	.2760
Lymphocytes (%)	0.1513 ± 0.027	0.1189 ± 0.042	.4792
d-ROM (U.Carr)	414.9 ± 15.82	352.4 ± 18.77	.**0087**
PAT (U.Carr)	2665 ± 133.5	2868 ± 160.9	.2500
OSI	92.25 ± 6.60	51.89 ± 6.45	.**0006**

**Figure 1.**
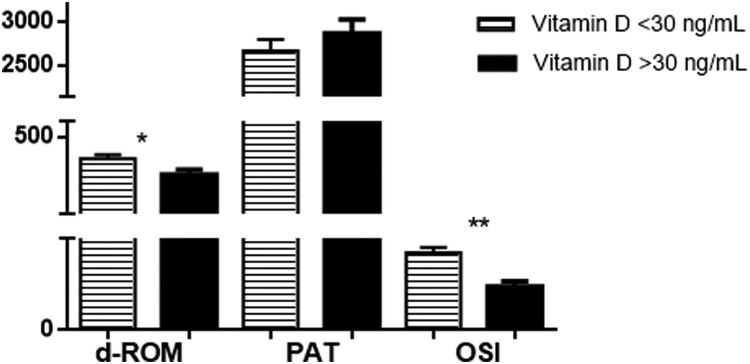
Graphical presentation of oxidative stress parameters for the two groups of COVID-19 patients with different vitamin D levels (measured as 25(OH)D). Results are shown as Mean ± SEM. *p < .05, **p < .01

An important finding of this study is that the oxidative stress was greater in vitamin D insufficient patients, as determined by significantly higher levels of d-ROMs (414.9 ± 15.82 U.Carr vs. 352.4 ± 18.77 U.Carr, *t*-test, *p* < .05) and OSI (92.25 ± 6.60 vs. 51.89 ± 6.45, *t*-test, *p* < .001), but without a difference in the antioxidant capacity between the two study groups (PAT = 2665 ± 133.5 U.Carr vs. 2868 ± 160.9 U.Carr, *t*-test, *p* > .05) (Figure 1).

Furthermore, we examined the correlations between serum vitamin D levels and selected laboratory parameters (d-ROM, OSI, LDH and D-dimer) with statistically significant differences between the two groups of COVID-19 patients divided according to their serum vitamin D levels at admission.

**Figure 2.**
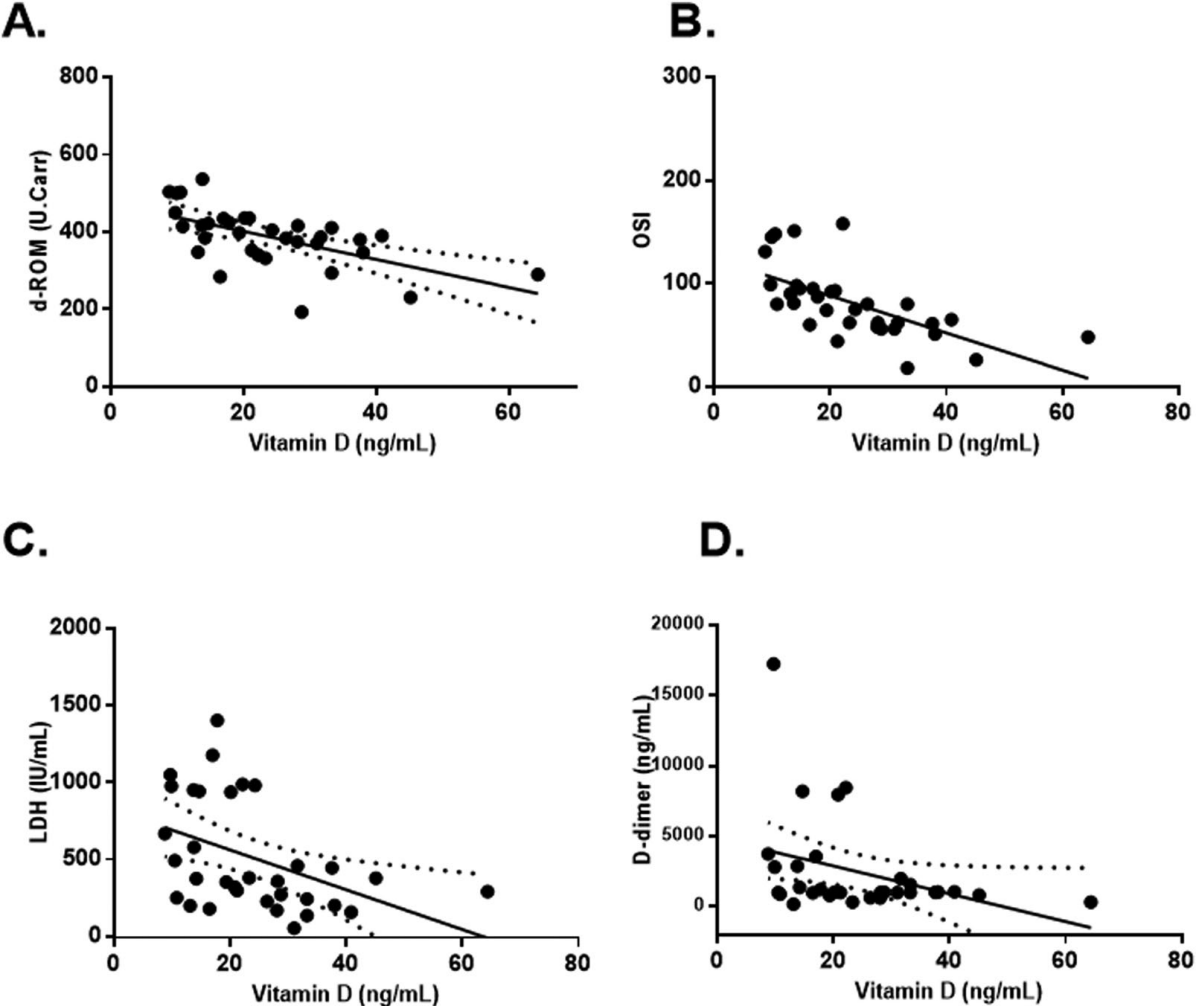
Linear correlations between serum vitamin D levels and d-ROM (A), OSI (B), LDH (C) and D-dimer (D) in COVID-19 patients. Vitamin D measured as 25(OH)D.

A significant inverse correlation was observed between vitamin D and d-ROM (*R*^2^ = 0.3482, *p* < .01), OSI (*R*^2^ = 0.4135, *p* < .001) and LDH (*R*^2^ = 0.1905, *p* < .05), whereas the correlation between vitamin D and D-dimers was found to be not statistically significant (*R*^2^ = 0.05263, *p* > .05) (Figure 2**)**.

We also took into consideration the outcome of the COVID-19 disease by subdividing the patients in each group as recovered and deceased. The mortality rate was higher (37.5%) in the group of patients with vitamin D levels below 30 ng/mL compared to 22.2% registered mortality rate in the vitamin D insufficient group (>30 ng/mL).

## 4. Discussion

The findings of our study indicate that severe COVID-19 patients have lower serum vitamin D levels and higher oxidative stress compared to the moderate group of patients when admitted to the hospital. Our results are in agreement with several other clinical studies [22, 23]. Panagiotou reported that vitamin D deficiency was more prevalent among patients requiring ITU (Intensive Therapy Unit) admission and proposed that vitamin D deficiency might be an under-recognized determinant for illness severity [24]. In the study of Macaya et al., vitamin D deficiency tended to predict an increased risk of developing severe COVID-19 after adjusting for age, gender, obesity, cardiac disease and renal disease [25]. Carpagnano et al. [26] determined that patients with severe vitamin D deficiency had a significantly higher mortality risk. In our study, the mortality rate was higher (37.5%) in the group of patients with lower vitamin D levels compared to 22.2% in the vitamin D replete group, but we were unable to detect a significant difference.

The immunomodulatory and anti-inflammatory effects of vitamin D are well known and elaborated [27–29]. Herein we show that COVID-19 patients with lower vitamin D levels have higher values of LDH (604.8 ± 76.98 IU/mL vs. 261.57 + 47.33 IU/mL, *t*-test, *p* < .05) at admission. LDH has been described to be increased during acute and severe lung damage, and elevated LDH values have been found in other interstitial lung infections [30, 31]. Additionally, the patients with vitamin D insufficiency had higher CRP levels at admission, which is a reliable marker of acute inflammation, lower platelet count and higher neutrophil to lymphocyte ratio (NLR) compared to the patients with vitamin D sufficient levels, but these changes were found to be not statistically significant (*p* > .05 for all three parameters), presumably due to small sample size. Among COVID-19 patients with lower serum vitamin D levels, we measured significantly higher D-dimer level (5978 ± 2028 ng/mL vs 977.7 ± 172 ng/mL, *t*-test, *p* < .05). Similar results were reported by Baktash et al. [32] where hospitalized older patients (> 65 years) with COVID-19 had vitamin D deficiency. Even though the herein results are descriptive and do not provide detailed view in the mechanism, one of the proposed mechanisms could be mitochondrial dysfunction associated with vitamin D deficiency. Namely, vitamin D can normalize mitochondrial dynamics with improving oxidative stress, cytokine production and pro-inflammatory state. Vitamin D has been demonstrated to down regulate the production of inflammatory cytokines (IL6, TNF-alpha) while increasing the inhibitory cytokines [33, 34]. Furthermore, vitamin D reduces activation of renin-angiotensin-aldosterone system (RAAS) and hence decreases ROS generation and improves the prognosis of SARS-CoV-2 infection.

Several experimental studies suggest that vitamin D insufficiency promotes a prothrombotic state, which could adversely influence the severity and outcome of COVID-19. Wu-Wong et al. [35] found that vitamin D receptor could play a role in atherothrombosis via regulation of plasminogen activator inhibitor-1 (PAI-1), thrombospondin-1 (THBS1) and thrombomodulin (TM) in human aortic smooth muscle cells. In one experimental study, vitamin D receptor knockout mice manifested significantly enhanced platelet aggregation and exacerbated multi-organ thrombus formation after exogenous lipopolysaccharide injection regardless of the calcemic conditions [36].

A valuable finding of this study is that the oxidative stress was greater in patients with lower vitamin D levels, as determined by higher values of d-ROMs (414.9 ± 15.82 U.Carr vs. 352.4 ± 18.77 U.Carr, *p* < .05) and OSI (92.25 ± 6.60 vs. 51.89 ± 6.45, *p* < .001), and with a significant inverse correlation between vitamin D and both parameters.

The beneficial effects of vitamin D are mediated mainly by modulating the expression of the endogenous antioxidant enzymes, such as monoamine oxidase and superoxide dismutase [37–41] and by adequate maintenance of the mitochondrial function [42, 43]. In addition, hypovitaminosis D may cause a decrease in the intracellular concentrations of glutathione, mediated by the enzyme gamma-glutamyl-transpeptidase and a limited ability to reduce the free radical levels [44]. COVID-19 and its complications have been associated with the oxidative stress in several review articles. The interaction between the virus and the host cells leads to loss of membrane integrity and mitochondrial dysfunction, which provokes an increased production of ROS [14–17, 45, 46]. The results from our study indicate a higher degree of oxidative stress in patients with a severe form of the disease compared to moderate COVID-19 patients (Table 2). Considering the above-elaborated vitamin D antioxidant actions, supplementation with this vitamin could considerably improve the clinical presentation of the SARS-CoV-2 infection.

The complex interplay of multiple causative mechanisms involved in pathophysiology of SARS-CoV 2 infection is challenging to elucidate. The presented data provide a justification to consider vitamin D as an important factor that could ameliorate disease severity through its anti-inflammatory, antithrombotic and antioxidant effects. Assessment of vitamin D status is an easy accessible and affordable laboratory tool in discriminating the patients’ potential of developing a severe form of the disease. Our findings suggest a benefit from vitamin D supplementation in the supportive treatment of SARS-CoV 2 infected patients, as well as in preventing occurrence of COVID-19 related complications.

The authors of this study acknowledge that the main limitation of this study is that it was conducted at a single centre with a relatively small number of patients. Due to work overload of the hospital team, as well as limited financial resources for the non-standard laboratory analyses, we were unable to include more subjects in the analysis.

Considering that several months are needed to achieve steady-state vitamin D levels, we focused only on the measurement of vitamin D levels at admission and did not obtain information about the supplemental therapy before hospitalization.

Therefore, the future research should be planned with bigger sample size and standardization of the patients according to their nutritional status, supplemental therapy and sunlight exposure as much as possible in order to further clarify the association between vitamin D and oxidative stress parameters in COVID-19.

## 5. Conclusion

Our results point to a link between increased oxidative stress, low vitamin D levels and disease severity in COVID-19 patients. In parallel to the immunization of the population, which aims on preventing of spreading the SARS-CoV-2 infection, further investigation should be focused on various therapeutic strategies that could influence the severity and the outcome of COVID-19. Among them, the benefit of vitamin D supplementation and potential therapies that reduce oxidative stress should be further assessed in large-scaled randomized controlled studies.

## Author contributions

All authors have accepted responsibility for the entire content of this manuscript and approved its submission.
